# Supplementary material for: End Sequence Analysis Toolkit (ESAT) expands the extractable information from single-cell RNA-seq data
Source: Genome Res. 2016 Oct;26(10):1397–410. doi: 10.1101/gr.207902.116 (PMC5052061; doi:10.1101/gr.207902.116)
Supplement: Supplemental Material [file supp_gr.207902.116_Supp_Materials.tar.gz › ESAT-esat_v0.1/doc/html/classbroad_1_1core_1_1datastructures_1_1_illegal_tree_operation_exception.html]

ESAT: broad.core.datastructures.IllegalTreeOperationException Class Reference


ESAT


- Main Page
- Related Pages
- Packages
- Classes
- Files

- Class List
- Class Index
- Class Hierarchy
- Class Members

All Classes Namespaces Files Functions Variables Enumerator Pages

- broad
- core
- datastructures
- IllegalTreeOperationException

Public Member Functions |
List of all members

broad.core.datastructures.IllegalTreeOperationException Class Reference

Inheritance diagram for broad.core.datastructures.IllegalTreeOperationException:

[legend]

Collaboration diagram for broad.core.datastructures.IllegalTreeOperationException:

[legend]

|  |  |
| --- | --- |
| Public Member Functions | |
|  | IllegalTreeOperationException (String msg) |
|  | |

## Constructor & Destructor Documentation

|  |  |  |  |  |  |
| --- | --- | --- | --- | --- | --- |
| broad.core.datastructures.IllegalTreeOperationException.IllegalTreeOperationException | ( | String | *msg* | ) |  |

---

The documentation for this class was generated from the following file:

- java/broad/core/datastructures/IllegalTreeOperationException.java


---

Generated on Fri Aug 15 2014 15:10:51 for ESAT by  

 1.8.7
